# Scientific Items

**Published:** 1886-08-20

**Authors:** 


### SCIENTIFIC ITEMS.

**Struck by a Meteor.**—A correspondent writes: "As a gentleman, a well known public official, was passing from St. James' Park into Pall Mall by the garden wall of Marlborough House, recently, at a quarter to 5 in the afternoon, he suddenly received on the right shoulder a violent blow, accompanied by a loud crackling noise, which caused him great pain and to stumble forward as he walked. On recovering his footing, and turning round to see who had so unceremoniously struck him, he found that there was no one on the pavement but himself and the policeman on duty at the park end of it. On reaching home the shoulder was submitted to examination, but nothing was at first discovered to account for the pain in it. But in a little while the servant who had taken away the coat to brush brought it back to point out that over the right shoulder the nap was pressed down flat in a long, straight line, exactly as if a hot wire had been sharply drawn across the cloth. The accident is therefore explained as having been caused by the explosion of a minute falling star or meteor. It is an unprecedented and most interesting occurrence, and deserves, I think, to be placed on public record."—*London Times.*

**Cyclamose—A New Sugar.**—Michan announces (*Chemical News*) the discovery by him of a new sugar. It exists in the tubeıs of *Cyclamen Europeum* and has been named "cyclamose." Cyclamose may be extracted from the tubers of the plant by digesting them for some days with alcohol of 80 per cent., concentrating and filtering the solution, precipitating the crude sugar with alcohol of 96 per cent. or over, and washing the product with the same strong spirit. The impure cyclamose is then dissolved in water, treated with milk of lime in excess, and the filtrate again precipitated with spirit. Redissolved in water, a current of carbon dioxide sets free the new sugar, and the filtered liquid evaporated *in vacuo* over sulphuric acid, leaves the cyclamose in a state of considerable purity. The formula of the new sugar is given as $C_{12} H_{22} O_{11}$. It has the peculiarity of being lævogyre, while all the other known sugars of the group are dextro-rotary to polarized light.—*Druggists' Circular.*

**Artificial Cocaine.**—Mr. Merck, of Darmstadt, according to a German journal, has succeeded in manufacturing cocaine from benzoyl-ecgonin, a body previously discovered by himself. He proceeds in the following manner: Several grams of benzoyl-ecgonin, with a slightly larger quantity of iodide of methyl and a little methyl alcohol, are heated in a tube to 100°. This mixture is digested in a water bath, to expel the undecomposed iodide of methyl alcohol. From the syrupy residue cocaine is extracted as a hydriodate. From this salt, pure cocaine, dissolving at 98°—the same as natural cocaine—is produced. The artificial substance is found to answer all tests.—*Popular Science News.*

**New Light on the Germ Theory.**—The second editorial of the New York *Medical Journal*, No. of March 6, consists of a running commentary on experiments lately conducted in Washington, on protective inoculation. Dr. Salmon, of that city, thinks that he may be able to throw some light on the rationale of protective inoculation; experiments were performed by him in conjunction with Dr. Theobald Smith, which we think, in their results, may modify very extensively the importance attached to formed germs in the role of conveying and propagating disease. Experiments were performed on pigeons with the contagium of swine plague. The gist of the experiment is contained in the following lines from the New York *Medical Journal:* "A ten minutes' exposure of liquid cultivations to a temperature of 58° C. (136.4°.) kills the microbe, but, according to the theory, leaves the liquid charged with the chemical products of its growth. This heated virus was used for the protective inoculation of pigeons. The virus which has not been mitigated invariably kills unprotected pigeons within twenty-four hours."

Thus the heated virus containing the dead microbes, was as efficient as ordinary virus containing live microbes. Then what gives disease ?—*Eastern Medical Journal.*

**A Few Remarks on Papayotin and Papain.**—From this paper we extract the following: "The digestive process of papain, which name has been given the dried milk juice, and of papayotin, differs from that of pepsin, rennet, and pancreatic juice, in that no acid is required to start the metamorphosis to peptone; and while papayotin does not act upon farinaceous food, its action upon all nitrogenous food is powerful. If gluten is digested over night with a solution of papain or papayotin it becomes nearly dissolved. As the papaw tree no doubt can be cultivated in Louisiana, it would doubtless prove of interest to the members of this association who dwell in country towns to experiment with the plant and prepare papain for which, probably soon, a larger demand may spring up."—*National Druggist.*

**The Chemical Barometer.**—The liquid in this weather-glass is made as follows: Take camphor 2 parts, sal ammoniac 1 part, salt petre 1 part, dissolve in alcohol, and add water until the camphor is partly precipitated, put in a bottle about ten inches long, cork well and seal with wax, and then perforate cork by burning through one small hole by means of a hot needle.

*Directions.*—If clear weather, precipitate remains at bottom. Change for storms: small crystals like stars will be seen floating in liquid; storm, precipitate rises till near the top; squall, long, hairy crystals will rise on the side from which the wind blows.—C. F, S.—*U. S. Medical Investigator.*

THE jointed rods which develop into chaplets, found in the blood passed from the bowels of persons having typhoid fever, are thought to be a species of spirillum.

